# Radiation Oncology

**DOI:** 10.1080/13577140120097076

**Published:** 2001-11


### RADIATION ONCOLOGY

### 062 Use of Intensity Modulated Radiotherapy (IMRT) in the Treatment of Sarcoma

Martin H Robinson[1], Barbara-Ann Millar[2], John Conway[3], Christopher M Bragg[1]
[1]*YCR Unit of Clinical Oncology, Weston Park Hospital,* [2]*Department of Clinical Oncology, Weston Park Hospital,* [3]*Department of Medical Physics, Weston Park Hospital*

*Objective:* Intensity modulated radiotherapy (IMRT) is an exciting new technique with the potential to improve radiotherapy treatment by reducing dose to normal structures and increasing dose to the cancer. The objective was to investigate the potential value of IMRT in the treatment of sarcoma. To compare normal tissue sparing and treatment volume coverage for both three dimensional conformal (3D-CRT) and IMRT techniques.
*Methods:* The diagnostic and planning images of 9 patients treated pre or post operatively for sarcoma (4 limb extremity, 3 pelvic, 1 nasal sinuses, and 1 trunk). Using radiotherapy planning computerised tomography (CT) images and diagnostic magnetic resonance imaging (Mm) and CT imaging for each patient, the gross original tumour, clinical target volume, and planning target volume were outlined along with normal tissue structures. Normal tissues were assigned appropriate tolerances for radiotherapy. Radiotherapy plans were then produced using conformal and IMRT planning techniques.
*Results:* Regret analysis, normal tissue complication probability and Conformity Index (CI) were used to compare the ability of each technique to optimise dose to tumour whilst sparing normal structures. Extremities: The CI was improved with IMRT in both PTV-I and PTV-II. In one case, the PTV-I with 3D-CRT gave a value of 0.14 improving with IMRT to 0.32 (5f-IMRT). PTV-I was also shown to improve with initial 3D-CRT value of 0.58, increasing to 0.84 (5f-IMRT). However, it was not possible to utilise all of the multiple fields in one case due to the field length being greater than 25 cm and therefore the maximum number of fields was limited to five. This is felt to have contributed to the poorer overall results for this case. In extremity sites, where the treatment volumes include considerable amounts of normal tissue, it was possible to limit the dose to bone and subcutaneous tissue using IMRT by up to 20%. Pelvic: In planning treatment for pelvic sarcoma again improvement in conformity was demonstrated with IMRT, especially when using greater than 5 fields. A deterioration in homogeneity and increase in under- and overdosing of both PTV-I and PTV-Il may have been due to a compromise in optimisation due to the position of the target relative to the surrounding normal tissue structure such as the rectum. Trunk and paranasal sinuses: In these cases, due to the site of the original tumour, margins were added to the original tumour volume and this was treated in a single phase. In the case of soft tissue sarcoma of the trunk, IMRT showed a considerable improvement in homogeneity, conformity index and underdosing. The conformity index increased from 0.52 (3D-CRT) to 0.85 (6f-IMRT). The majority of organs at risk demonstrated a consistent reduction in maximum dose received, e.g. the heart received 19.7 Gy (3D-CRT) compared to 6.2 Gy (7f-IMRT). There was a slight increase in the dose received by the spinal cord but this remained below 8 Gy in all cases. In the case of the osteosarcoma arising in the paranasal sinuses, again conformity was improved but at the expense of homogeneity due to the close proximity of the organs at risk to the tumour volume. It was possible to reduce the mean dose to organs at risk, in particular the lens and the eye. In the right lens, a complication probability of 100% with 3D-CRT was reduced to 75% and in the left lens it was reduced from 100% to 30%. Reviewing the tumour control probability (TCP) for IMRT, there was a trend towards improvement in TCP with an increased number of fields and this was generally mirrored in the regret scores and conformity index.
*Conclusion:* IMRT has the potential to improve quality of radiotherapy in sarcoma practice. This benefit appears to vary with tumour site.

### 070 Indications for Radiotherapy After Near Complete Resection (Microscopic Residual Diesease, IRS Group II) in Rhabdomyosarcoma: A Report from the German Soft Tissue Sarcoma Study Group CWS

Ewa B Koscielniak[1], Robert Knietig[1], Bernhard F Schmidt[2], Denise Kunz[1], Adrian Mattke[1], Joern Treuner[1]
[1]*Dept.of Pediatric Hematology/Oncology, Olga Hospital,* [2]*Dept.of Radiotherapy, Katharinen Hospital*

*Objective:* To evaluate the outcome of patients with localised, primary resected rhabdomyosarcoma (RMS) with microscopic residual disease, treated with multiagent chemotherapy, with or without local radiotherapy.
*Methods:* One hundred thirteen patients with near complete resected localised RMS (IRS Group II) were further treated with chemotherapy (vincristine, cyclophosphamide or ifosfamide, dactinomycin ± doxorubicin ± VP16) on German Soft Tissue Sarcoma Studies CWS-81 to CWS-96 between 1981-1997. Sixty two patients (54%) also received radiotherapy (RTX). The RTX was recommended for patients with microscopic disease after second look surgery (CWS-81), for patients with extremity and parameningeal primary tumors and those with bone erosion at presentation (CWS-81 to CWS-91), for patients with alveolar RMS (CWS-91) and for all Group II patients in the CWS-96. In the CWS-81 Study doses between 40-50 Gy (1 x 1.8 Gy/day) were administered. Since 1986 the CWS-Group recommend an accelerated hyperfractionated RTX (2 x 1,6 Gy daily) with doses between 32-54 Gy.
*Results:* Fourteen irradiated (22%) and eighteen (35%) non-irradiated patients relapsed (p=0.1). 78% (25/32) of failure sites were local, 6 % were regional and 15% were distant. The proportion of patients who were irradiated differed slightly between the Studies (43-77%). 61% of patients younger than 3yrs of age were irradiated in contrast to 78% of patients older than 3yrs. The 5 yrs event-free survival (EFS) and 5 yrs overall survival (S) were 80±9% and 85± 8% (CWS-81), 71±7% and 89±5% (CWS-86),73±8% and 93±5% (CWS-91) respectively. The median irradiation dose was 40 Gy (CWS-81), 32Gy (CWS-86), 36,5Gy (CWS-91) and 44Gy (CWS-96). In the multivariate analysis no differences in EFS were observed by tumor size, site, age, alveolar histology or by whether or not the patient received RTX. However, the alveolar histology was shown to be predictive of overall survival (p=0.01).
*Conclusion:* Patients with group II RMS have a very good prognosis when treated with adjuvant multiagent chemotherapy with or without irradiation. RTX was not found to benefit significantly the EFS and S of patients with embryonal RMS. However, the addition of RTX was associated with improved survival in patients with alveolar RMS.

### 092 High Resolution, Intensity Modulated Radiation therapy (IMRT) for Retroperitoneal Soft Tissue Sarcoma (RPS)

Tara Haycocks[1], Valerie Kelly[1], Mohammad Islam[1], Brian O'Sullivan[1], Carol Jane Swallow[2], Charles Nicholas Catton[1]
[1]*Radiation Medicine Program, Princess Margaret Hospital and The University Of Toronto,* [2]*Department of Surgical Oncology, Princess Margaret Hospital and The University of Toronto*

*Objective:* RPS are large tumors that are often positioned between critical radiosensitive structures, and which present challenging

DOI: 10.1080/13577140120097076

treatment planning scenarios. Bowel may be adequately excluded from the high-dose radiation volume with pre-operative conformal therapy, but liver, kidney and cord within or adjacent to the clinical target volume (CTV) often compromise radiation delivery. This study compares IMRT to conventional conformal techniques with regard to the radiation dose profile to the CTV and adjacent critical structures.

*Methods:* 9 patients were referred for pre-operative radiotherapy of RPS. 6 presented with right-sided tumors, 3 with left-sided tumors. 5 right-sided tumors were located in both the upper and lower quadrants of the abdomen, 1 in the lower quadrant only. All left-sided tumors were located in the lower quadrant. The gross tumor volume (GTV), CTV, liver, cord, bowel and the non-involved kidney were contoured throughout the region of interest. The CTV encompassed the GTV with a 1.5 to 5 cm margin, as determined by the clinical situation. Patients were prescribed 45 Gy in 25 fractions over 5 weeks. A forward conformal plan and an inverse IMRT plan were generated for each patient. Co-planar conformal distributions employed 2 to 4 beams with 6 or 25MV photons. IMRT beam fluence was optimized by the Helios inverse planning system for 10MV photons, using 6 to 9 co-planar beams and a Varian 120-leaf dynamic multi-leaf collimator. The conformity index (CI), and dose-volume histograms (DVH) of conformal and IMRT plans were compared for each patient with regard to CTV, liver, cord, bowel, and the contralateral kidney. The statistical significance of differences in DVH were determined with the two-tailed t-test.

*Results:* Mean volumes of the GTV and CTV were 2042cc (range 93–4668cc) and 4042cc (range 478 – 6532cc) respectively. Dose homogeneity is comparable for both treatment systems. Mean variance within the CTV for conformal plans was +6% to –9% and was +7 to –7% for IMRT plans. The CI of the IMRT plans was significantly smaller ($p < 0.05$). Mean for IMRT was 1.1 (range 1.0 – 1.2), and 2.1 (range 1.2 – 5.4) for conformal plans. IMRT significantly reduced mean %bowel volume within the 40 Gy isodose (17% vs 30%, $p<0.05$) and reduced the maximum dose given to the 25% volume of bowel (30Gy vs 41Gy, $p < 0.05$). IMRT significantly reduced the maximum dose to the liver for right upper quadrant tumors ($p<0.05$).

*Conclusion:* Patients with RPS presented for pre-operative RT with huge tumors adjacent to critical uninvolved structures. The improved CI with IMRT reduced the dose to uninvolved liver and bowel. This may result in overall reduced toxicity and for some patients with right upper quadrant tumors, IMRT represented the only opportunity for safe radiation delivery to an adequate CTV. Pre-operative IMRT for RPS offers an opportunity to improve the CTV coverage and/or increase the dose to 50 Gy or higher, while meeting the dose constraints of adjacent normal structures. This may result in less toxicity, improved local control, and improved disease-free survival.

### 102 Irradiation of the Dura Intra-Operatively by Customized 90Y Plaques

Herman Day Suit[1], George T.Y. Chen[1], Thomas Mauceri[1], John Monro[2], Francis J. Hornicek[1], Frank Pedlow[1], Thomas F. DeLaney[1]

*[1]Depts of Radiation Oncology and Orthopaedic Surgery, Massachusetts General Hospital, Harvard Medical School, [2]Implant Sciences, Inc.*

*Objective:* Objective. A high proportion of tumors of the vertebral bodies which have extended into the vertebral canal impinge on the dura and displace it and in most such instances the cord. The tumor will be in direct contact and have invaded the dura on a microscopic basis. In addition to the intra-canal extension there is accompanying extension into the paravertebral tissues in many of these patients. The residual tumor cells on the dura are judged to be a contributing factor in the high incidence of local failures. External beam techniques do not permit adequate doses to the dura and respecting the tolerance limits of the spinal cord. Hence, the need for dose to be given by high technology procedures directly to the dura as a component of treatment of this category of patients.

*Methods:* Our management strategy is pre-operative radiation in the amount of 50 Gy by intensity modulated X ray or proton beam technique, This results in about approximately 40 Gy to the cord center and marginally higher to the dura. Next, there is resection of the vertebral body [s] and any soft tissue mass. At that point the dura is cleanly exposed but judged to be microscopically positive. A special 90Y foil had been prepared to fit the affected area of the dura. The physical characteristics are: maximum electron energy is 2.3 MeV, dose at 2.5 mm is approximately 20%. The foil is embedded in a plastic applicator with 0.5 mm between foil and the surface, e.g., dura. This system is almost radiation exposure free for the staff. The applicator is applied to the area of concern of the dura. A single dose of 15 Gy requires 10-15 min. Following the intra-operative irradiation, the patient is closed. Post operatively, the treatment is completed. Chemotherapy is administered to patients with high grade large sarcomas.

*Results:* Results. We have progressed through 3 plaque designs. Four patients have been treated to date; for one, the 90Y foil has been utilized. The other 3 patients were treated by 192Ir plaque or a plaque containing 90Y liquid. This foil is vastly superior to the earlier two models. The applications have been without complications and have required ~25 additional minutes.

*Conclusion:* Conclusions. The 90Y foil system is a technically an easy plaque to utilize and the dose distribution is very attractive for inactivation of tumor cells on the dura.

### 103 Neoadjuvant Chemotherapy and Radiotherapy for Large Extremity Soft Tissue Sarcomas

Thomas F. DeLaney[1], Ira J. Spiro[1], Herman D. Suit[1], Mark C. Gebhardt[2], Francis J. Hornicek[2], Henry J. Mankin[2], Andrew L. Rosenberg[3], Fariba Miryousefi[1], Marek Ancukiewicz[1], David C. Harmon[4]

*[1]Dept. of Radiation Oncology, Massachusetts General Hospital, [2]Dept. of Orthopedic Surgery, Massachusetts General Hospital, [3]Dept. of Pathology, Massachusetts General Hospital, [4]Section of Hematology/ Oncology, Massachusetts General Hospital*

*Objective:* Treatment of extremity soft tissue sarcomas (STS) yields excellent local control but distant failure is common with large, high-grade tumors. A regimen of pre-operative chemotherapy consisting of Mesna, Adriamycin, Ifosfamide, and Dacarbazine (MAID) interdigitated with radiotherapy followed by resection and postoperative chemotherapy +/- radiotherapy was designed to improve treatment outcome. We report mature outcome data on 48 treated patients and compare with an historical matched control patient population.

*Methods:* Adult patients with high-grade extremity STS 8 cm or larger were treated with three cycles of preoperative chemotherapy combined with 44 Gy of radiotherapy followed by surgery. Three cycles of postop MAID were planned. For patients with positive surgical margins, 16 Gy was delivered postoperatively.

*Results:* 48 M0 patients received MAID protocol treatment and outcome was superior to 48 historical control patients. Respective five-year actuarial MAID and control patient figures for local control are 92% and 86% (p=0.1155), freedom from distant metastases are 75% and 44% (p=0.0016), disease-free survival are 70% and 42% (p=0.0002), and overall survival are 87% and 58% (p=0.0003). Acute hematologic toxicity in the MAID group included febrile neutropenia in 7 patients (15%). RTOG grade 3 acute skin toxicity occurred in 14 patients (29%). One MAID patient developed late fatal myelodysplasia.
*Conclusion:* Following aggressive chemoradiation and surgery, these patients show a significant reduction in distant metastases with a highly significant gain in disease-free and overall survival when compared to historical control. Based on this experience, the Radiation Therapy Oncology Group has conducted a multi-institutional trial.

**109 Radiation Induced Leiomyosarcoma: A Report of 3 Cases**

Bruce E Brockstein[1], Arno Mundt[2], Daniel J Haraf[2], Anthony Montag[2]
*[1]Evanston Hospital, Northwestern University, [2]University of Chicago*

*Objective:* To report a series of 3 cases demonstrating that leiomyosarcoma may be a radiation induced malignancy.
*Methods:* Presentation of a series of three cases.
*Results:* A variety of solid tumors have been reported to occur within the radiation fields of radiation administered for both benign and malignant conditions. A variety of bone and soft-tissue sarcomas have been reported to occur in this setting. Leiomyosarcoma, however, has rarely been reported as a secondary malignancy following radiotherapy. We report on three such cases. CASE #1: A 71 year old man was treated 10 years before presentation with a low anterior resection for rectal cancer, followed by 5-FU chemotherapy and radiation. Pulmonary metastases of unknown primary site were histologically identified as a high grade leiomyosarcoma. Subsequently, the primary site was identified as a perirectal mass extending into his left buttock. The patient was treated palliatively. CASE #2 A 60 year old man was treated over a 30 year period for relapsing midline destructive disease with surgery, antibiotics, and chemotherapy. In addition, he was treated with radiation to the mid-face 27 and 14 years prior to the current presentation. A small mass appeared beneath the left eye and prompted a CT scan of the face and sinuses, demonstrating a large left facial mass with involvement of the maxillary sinus and orbit. A biopsy revealed a high grade leiomyosarcoma. The patient was treated with three cycles of adriamycin and ifosfamide chemotherapy. Resection of residual tissue revealed only microscopic foci of leiomyosarcoma. He received 2 more cycles of adriamycin and ifosfamide, and remains well after a year. CASE #3- A 76 year old woman was treated 12 years prior for a T2N0M0 squamous carcinoma of the epiglottis with laser excision, and 10 years prior with chemoradiation after recurrence of her cancer. The patient remained well, but was found to have a large, obstructive tracheal mass when she presented with stridor. Biopsy revealed a high grade leiomyosarcoma. The patient received one course of palliative chemotherapy before succumbing to her disease.
*Conclusion:* These 3 cases demonstrate that leiomyosarcoma may occur as a consequence of prior radiotherapy. Concomitant chemotherapy was given in all 3 patients reported here, and may play a role in the pathogenesis. Typical onset occurs after 10 years, and the clinical course may be variable.

**114 Marrow Stromal Cell Transplant Improves Wound Healing Following Radiation**

Robert Stuart Bell, O'Sullivan Brian, Hill Richard, Armand Keating
*Princess Margaret Hospital, University of Toronto, Toronto, Canada*

*Objective:* We have previously shown that intra-dermal injection of autologous dermal fibroblasts partly reverses the effect of radiation on healing of a rat surgical model. In the current experiments, we have tested the effect of syngeneic marrow stromal cells transplantation on healing of irradiated deep and superficial wounds.
*Methods:* Lewis rats received bilateral buttock irradiation (18 Gy single fraction) followed by creation of bilateral buttock incisions three weeks after radiation. At the time of surgery, one side received 106 pooled syngeneic marrow stromal cells. Twenty-one days following surgery, mechanical wound testing was performed.
*Results:* The dermal injection of marrow stromal cells resulted in marked increases in breaking strength when compared to the control group breaking strength 338.5 g + 39.9g vs. 187.1g + 12.0g; p< 0.01 (Wilcoxon sign rank test).
*Conclusion:* Many patients may benefit from radiation prior to surgery, but wound healing complications are common following preoperative radiation. We have previously shown that dermal fibroblast transplantation into the radiated wound improves wound mechanical characteristics. Marrow stromal cells have a possibly greater effect on radiated wounds than dermal fibroblasts.

**129 A Comparison of Lower Extremity Functional Outcome Following Limb Perfusion, Pre- or Post-Operative Radiotherapy for Soft Tissue Sarcoma**

Peter Hohenberger[1], Jay Wunder[2], Anja Herrmann[1], Anthony Griffin[2], Robert Bell[2], Brian O'Sullivan[3], Charles Catton[3], Aileen Davis[4]
*[1]Robert-Roessle Hospital and Tumor Institute at the Max-Debruck Center for Molecular Medicine, [2]Mount Sinai Hospital, [3]Princess Margaret Hospital, [4]Toronto Rehabilitation Institute*

*Objective:* The disability outcomes of patients with lower extremity soft tissue sarcoma (STS) treated with limb sparing surgery and adjuvant therapy of intra-arterial limb perfusion (ILP) or preoperative external beam radiotherapy (preop) or post-operative external beam radiotherapy (postop) were compared.
*Methods:* 29 patients in each of the three treatment groups were matched for age, anatomical tumour site, tumour size and months of functional follow-up. Baseline sample characteristics (age, gender, primary vs. recurrent tumour) and variables known to impact on functional outcome (motor nerve sacrifice, bone resection, complications, vascular reconstruction) were compared to ensure group similarity. The primary outcome, the Toronto Extremity Salvage Score (TESS), was evaluated among the three groups using a paired t-test and then regression analysis.
*Results:* There were no significant differences among the groups on any of age (group mean range 44 to 47), tumour size, anatomical location, months of functional follow-up (group mean range 21 to 23 mos). Although not significantly different, 11 of the ILP group presented with recurrent tumour as compared to 3 in the preop and 2 in the postop groups. There were no statistically significant differences in the number of cases with bone, major motor nerve, or vessel resection or in wound complications in the three groups. The ILP group had a lower TESS score than the postop rads group (p=0.005); TESS means of 80.8 vs. 91.5 vs. 85.6 for the ILP, preop and postop groups respectively. In a model evaluating gender and treatment group, gender was a significant predictor of the TESS (men mean of 87.1 vs women mean of 75.7, p=0.005) but treatment group was not significant (p=0.144). Use of pain medication was the same in all groups (6, 6 and 5 cases) but 5 in the ILP group used an ambulatory aids as compared to 2 and 1 in the preop and postop rads groups. At last follow-up 4 ILP patients and 1 postop case had developed local recurrence. 9 of the ILP, 6 of the preop and 4 of the postop group had metastatic disease.

*Conclusion:* STS patients undergoing ILP, preop or postop radiotherapy report relatively high levels of function with the least disability reported in the preop radiotherapy group. In these data, women have lower scores than men, particularly in the ILP treatment group.

